# Modulating Benign Prostatic Hyperplasia Through Physical Activity—The Emerging Role of Myokines: A Narrative Review

**DOI:** 10.3390/medicina61081362

**Published:** 2025-07-28

**Authors:** Saad Alshahrani

**Affiliations:** Department of Surgery, Division of Urology, College of Medicine, Prince Sattam bin Abdulaziz University, Alkharj 11942, Saudi Arabia; s.alshahrani@psau.edu.sa

**Keywords:** benign prostatic hyperplasia, exercise, myokines, inflammation, prostate health

## Abstract

Benign prostatic hyperplasia (BPH) is a multifactorial condition that is highly prevalent and affects aging males. It frequently results in lower urinary tract symptoms (LUTS) and a reduced quality of life. While hormonal dysregulation and chronic inflammation have long been implicated in BPH pathogenesis, recent evidence highlights the role of physical activity in modulating prostate health. In this narrative review, evidence from quantitative studies examining the effect of exercise on BPH risk and symptom severity was first synthesized. Collectively, these studies suggest that regular physical activity is associated with a lower incidence and reduced progression of BPH. The potential mechanisms through which exercise may exert protective effects on the prostate were then explored. These include modulation of sympathetic nervous system activity, alterations in hormonal profiles (e.g., testosterone and insulin), suppression of chronic inflammation and oxidative stress, and the promotion of autophagy within prostatic tissue. Central to these mechanisms is the role of myokines—signaling molecules secreted by skeletal muscle during exercise. Key myokines, such as irisin, interleukin-6 (IL-6), brain-derived neurotrophic factor (BDNF), and myostatin, are reviewed in the context of prostate health. These molecules regulate inflammatory pathways, metabolic processes, and tissue remodeling. For instance, exercise-induced reductions in myostatin are linked to improved insulin sensitivity and decreased fat accumulation, while elevated irisin and BDNF levels may exert anti-inflammatory and metabolic benefits relevant to BPH pathophysiology. Although direct causal evidence linking myokines to BPH is still emerging, their biological plausibility and observed systemic effects suggest a promising avenue for non-pharmacological intervention. Future research should focus on identifying the specific myokines involved, elucidating their molecular mechanisms within the prostate, and evaluating their therapeutic potential in clinical trials.

## 1. Introduction

Benign prostatic hyperplasia (BPH), a non-malignant enlargement of the prostate gland, remains one of the most prevalent urological conditions affecting aging men worldwide [1,2]. Characterized by lower urinary tract symptoms (LUTS), such as urinary frequency, nocturia, and incomplete bladder emptying, BPH significantly impairs quality of life and imposes a growing socioeconomic burden on aging populations [3]. Traditionally, the pathophysiology of BPH has been attributed to hormonal imbalances, particularly androgenic and estrogenic influences, local inflammation, and stromal–epithelial interactions within prostate tissue [4]. However, recent epidemiological and mechanistic research has started to challenge this isolated organ-based perspective, shedding light on the role of systemic metabolic and inflammatory factors in the initiation and progression of prostatic overgrowth [5].

A growing body of evidence suggests that skeletal muscle functions not only as a mechanical organ but also as an endocrine tissue, capable of influencing distant organs through the release of myokines during physical activity [5,6]. These signaling molecules, such as irisin, interleukin-6 (IL-6), myostatin, and brain-derived neurotrophic factor (BDNF), exert autocrine, paracrine, and endocrine effects, contributing to systemic anti-inflammatory and metabolic benefits [7,8,9,10]. While their role has been explored in various tissues, the potential implications of myokine-mediated crosstalk with the prostate remain under-investigated in the context of BPH. Alongside this emerging interest in muscle–prostate communication, established evidence highlights both non-modifiable (e.g., age and race) and modifiable (e.g., diet and physical activity) risk factors for BPH [11,12,13]. In particular, physical activity has been consistently associated with a reduced risk of BPH and LUTS [14]. This protective association is thought to be mediated through several physiological mechanisms, including attenuation of sympathetic nervous system activity, hormonal modulation, suppression of inflammation and oxidative stress, and the induction of autophagy within prostatic tissue [15]. Furthermore, exercise-induced myokines may contribute to these effects by influencing inflammation and tissue remodeling within the prostate [7,8,9,10].

The objective of this narrative review is to investigate the diverse mechanisms through which exercise influences BPH to bridge the gap between exercise physiology and urological health. This review highlights the potential synergistic effects of these factors on the progression of BPH by synthesizing the existing evidence on the modulation of hormonal balance, reduction in inflammation, enhancement of tissue remodeling, and promotion of autophagy through regular physical activity. Furthermore, the potential crosstalk between the prostate gland and skeletal muscle is examined and discussed, with a focus on the role of myokines in this process.

## 2. Materials and Methods

This review aimed to synthesize findings from both clinical and experimental studies to describe how regular exercise and molecular mediators, particularly myokines, may influence the development and progression of BPH. The review followed a structured, thematic approach to ensure comprehensive coverage and critical appraisal of the existing literature.

The review began by identifying the central research question: How does physical activity, via the secretion of myokines and associated physiological mechanisms, influence prostate health, particularly in the context of BPH? To address this, a literature search was conducted using major scientific databases, including PubMed, Scopus, and Web of Science, up to April 2025. Search terms included combinations of “benign prostatic hyperplasia”, “BPH”, “exercise”, “physical activity”, “myokines”, “skeletal muscle”, “inflammation”, “autophagy”, “oxidative stress”, “hormonal changes”, and “sympathetic nervous system”. Only peer-reviewed articles published in English were included.

Relevant studies were screened by title and abstract, and full texts were retrieved for detailed evaluation. The inclusion criteria encompassed original research articles (both human and animal studies), reviews, and mechanistic studies that addressed the impact of exercise or myokines on prostate health or BPH-related pathways. Excluded were articles that did not address either BPH or the role of physical activity/myokines or were editorials, commentaries, or conference abstracts without full data.

Data from selected studies were thematically organized under key mechanisms through which exercise may influence BPH, including modulation of inflammation, hormonal profiles, sympathetic nervous system activity, oxidative stress, and autophagy. Particular emphasis was placed on the role of specific myokines, such as irisin, IL-6, BDNF, and myostatin. The review also critically examined the strengths and limitations of available evidence and highlighted areas for future investigation.

## 3. Results and Discussion

### 3.1. Etiology and Risk Factors of BPH and Potential Role of Physical Activity

**Figure 1:**
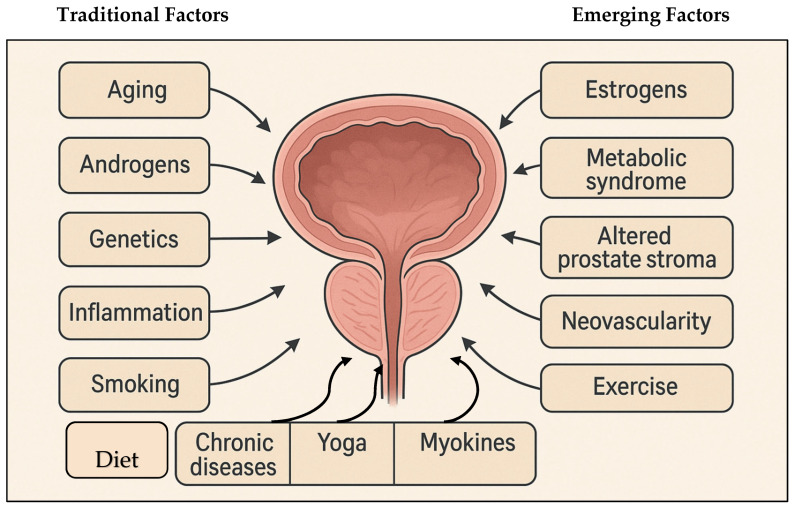
A schematic diagram illustrating traditional and emerging factors in BPH pathogenesis.

While the precise etiology of BPH remains incompletely understood, its development is known to involve a multifactorial interplay of hormonal, metabolic, and lifestyle factors [11]. Hormonal dysregulation, particularly imbalances in androgen and estrogen signaling, as well as overexpression of epithelial growth factors and steroid hormone receptors in both stromal and epithelial prostatic compartments, plays a central role in the pathological tissue remodeling seen in BPH [11,16]. One critical mediator is dihydrotestosterone (DHT), which disrupts the balance between cellular proliferation and apoptosis in prostatic tissues, contributing to nodular hyperplasia [17,18]. Beyond hormonal regulation, non-modifiable risk factors, such as age, race, and genetic predisposition, along with comorbid conditions, including diabetes mellitus, cardiovascular disease, and hypertension, have been consistently associated with increased BPH prevalence [3,19]. Modifiable lifestyle factors—particularly diet, physical activity, smoking, and alcohol intake—also contribute significantly to the disease’s pathogenesis [11,12,20], as shown in Figure 1 below.

The established cardiovascular and metabolic benefits of regular aerobic exercise suggest a biologically plausible protective mechanism against BPH [21,22,23]. The clinical management of BPH typically involves a range of therapeutic interventions, including the use of α-blockers, 5α-reductase inhibitors (such as finasteride and dutasteride), surgical procedures, or a combination of these approaches [24,25,26]. In addition to conventional treatments, various non-pharmacological interventions have shown potential in alleviating symptoms associated with BPH. These include pelvic floor muscle training, Pilates, yoga, whole-body vibration therapy, diaphragm and abdominal muscle exercises, and micturition interruption techniques, as well as complementary approaches such as acupuncture and auriculotherapy. Collectively, these interventions may contribute to symptom relief and improved quality of life in individuals with BPH [27].

A sedentary lifestyle is a recognized risk factor for a multitude of chronic diseases, including coronary artery disease, stroke, type 2 diabetes, osteoporosis, and certain types of cancer [28,29]. Conversely, adopting a physically active lifestyle has been shown to prevent, alleviate, and slow the progression of conditions such as metabolic syndrome [30]. Exercise may help regulate sympathetic nervous system tone, reduce systemic inflammation, and influence hormonal and growth factor signaling involved in prostatic enlargement [31,32]. In older men, the implementation of regular physical activity represents a potentially cost-effective and socioeconomically advantageous strategy for reducing BPH risk, possibly offering advantages over pharmacological or surgical interventions [33,34].

The well-documented health benefits of physical activity suggest a potential protective effect against the development and progression of BPH and its associated LUTS [33,34]. Proposed mechanisms underlying this protective role include a reduction in prostate size, a decrease in sympathetic nervous system activity, and the attenuation of systemic inflammation [13,31]. Some research has indicated that engaging in physical activity can alleviate existing BPH/LUTS symptoms [32]. However, a significant portion of these studies have relied on questionnaires to assess physical activity levels in individuals already diagnosed with BPH or LUTS. This methodological approach introduces the potential for recall bias, where individuals may inaccurately remember their past activity levels, and reverse causation, where the presence of LUTS-related discomfort might lead to a reduction in physical activity, thereby confounding the observed associations.

Consequently, there is a relative paucity of research specifically designed to investigate the prospective association between physical activity and the initial onset of BPH, mitigating the risks of recall bias and reverse causality. The findings from these etiological analyses remain inconclusive, with some studies failing to demonstrate a significant relationship between exercise and the development of BPH [35,36,37]. Table 1 synthesizes current epidemiologic evidence regarding the relationship between physical activity and the risk or progression of BPH/LUTS, clarifying these variable findings within a unified framework.

### 3.2. The Complex Relationship Between Physical Activity and Benign Prostatic Hyperplasia: A Review of Epidemiological Studies

Numerous epidemiological studies have examined the association between physical activity and the risk or progression of BPH and its accompanying LUTS. While findings vary, a substantial body of evidence supports an inverse relationship, suggesting that physical activity may play a protective role in BPH pathogenesis. However, variability in study designs, population characteristics, and methods of measuring exercise contribute to inconsistent observations across the literature.

A landmark prospective cohort study by Platz et al. serves as a cornerstone in this field [38]. Initiated in 1986, the study tracked 30,634 men aged 40–75 years over multiple follow-up intervals. BPH diagnosis was based on the following three criteria: surgical intervention, clinical symptoms, or a positive digital rectal examination [38]. Exercise levels were quantified using weekly metabolic equivalent of task hours (MET·hr/wk). The study revealed a significant inverse association: men in the highest quintile of physical activity (≥33.8 MET·hr/wk) exhibited a 25% lower odds ratio (OR = 0.75) of requiring BPH surgery compared to those in the lowest quintile (0.1–3 MET·hr/wk), after adjusting for several confounding factors [38]. This finding supports a dose–response relationship, indicating that increased physical activity may lower the clinical severity of BPH. Similarly, a 2001 cohort study by Meigs et al. followed 1019 men for an average of 9 years and assessed exercise frequency using a questionnaire [39]. High levels of physical activity were associated with a cross-sectional odds ratio of 0.5 for BPH compared to inactivity, indicating a halving of the odds of BPH in more physically active men, even after adjusting for factors such as age and waist-to-hip ratio [39]. These data re-enforce the hypothesis that regular exercise may directly influence prostate health, potentially through biological mechanisms, such as reduced sympathetic tone, improved hormonal balance, and attenuation of systemic inflammation.

A 2008 study by Williams involving a large cohort of 28,612 men between 1991 and 2002 demonstrated a clear dose-dependent inverse association between both running distance and exercise intensity and the risk of developing BPH [44]. Notably, the protective effects were strongest in those exceeding standard physical activity guidelines, suggesting that higher-than-recommended levels of endurance exercise may confer additional benefits [44]. In a study of 1033 Italian men with urinary disorders, Prezioso et al. observed that individuals who regularly engaged in physical activity exhibited significantly smaller prostate volumes and lower International Prostate Symptom Scores (IPSS) [40]. Furthermore, physically active patients showed a reduced prevalence of specific LUTS symptoms, including incomplete emptying, intermittent flow, and urinary frequency [40]. Rohrmann et al. conducted a case–control design with 2797 men experiencing LUTS. Their analysis revealed that men who engaged in higher levels of leisure-time physical activity had a 52% reduced risk of LUTS (OR = 0.48) compared to inactive individuals [42]. However, no clear dose–response trend was observed, suggesting that even moderate levels of exercise may be sufficient for symptomatic benefit. In another Italian study, Dal Maso et al. assessed 1369 men and found that the odds of BPH were consistently lower in men with higher levels of both occupational and recreational physical activity, with adjusted ORs of 0.6 to 0.7 across different age strata (15–59 years), indicating a potentially protective effect of sustained physical activity throughout the life course [36]. Similarly, Lagiou et al. evaluated occupational physical activity in 184 Greek men and found that those classified as having high physical activity at work had a significantly reduced odds of BPH (OR = 0.59) compared to men in the low-activity category [45]. These findings collectively reinforce the hypothesis that sustained physical activity, regardless of setting, may lower BPH risk by influencing systemic and local biological processes.

More recent research has focused on the impact of physical activity on specific BPH symptoms and overall risk. Wolin et al. found that in a cohort of 4710 BPH patients, engaging in at least one hour of weekly physical activity was associated with 13% lower odds of nocturia (OR = 0.87) and a substantial 34% lower odds of severe nocturia (OR = 0.66) [47]. This highlights a potential protective effect of even modest physical activity against bothersome BPH symptom. Further emphasizing the importance of lifestyle factors, a domestic study by Lee et al. in 582 men with BPH symptoms indicated that reduced sedentary time, specifically, played a more prominent role than overall exercise volume in mitigating the risk of BPH. Men who sat for more than 7 h daily had 72% higher odds of BPH (OR = 1.72) compared to those who sat for less [46]. This finding suggests that prolonged sitting, irrespective of structured exercise, may be an independent risk factor for BPH development or exacerbation. Complementing these observational insights, broader evidence from a systematic review and meta-analysis comprehensively suggests that engaging in moderate-to-vigorous physical activity could lower the risk of developing BPH or LUTS by up to 25% compared to leading a sedentary lifestyle [14]. This meta-analysis consolidates evidence from multiple studies, providing stronger support for physical activity as a preventive measure against BPH and its associated lower urinary tract symptoms.

However, not all studies have shown a clear inverse relationship between physical activity and BPH outcomes, highlighting the complexity of this association. Hong et al. found no overall difference in BPH risk between active and inactive men; however, their cross-sectional study unexpectedly revealed that moderate exercise was associated with an increased risk [43]. This particular finding warrants further investigation to understand potential confounding factors or unique population characteristics. Lacey et al. found no significant association between occupational physical activity and histologically confirmed BPH in a Chinese cohort [37], suggesting that the type or intensity of physical activity, or population-specific factors, may influence outcomes. Joseph et al. initially observed an inverse relationship between exercise and LUTS severity in African American men; however, this association was attenuated and, ultimately, disappeared after adjusting for key confounding factors, such as age, BMI, and socioeconomic status [41]. This indicates that while exercise might appear beneficial in unadjusted analyses, other variables may be the primary drivers of LUTS severity in this population. Similarly, Kristal et al. found no significant correlation between light, moderate, or high levels of physical activity and the incidence of BPH in a 7-year cohort study [35], further underscoring the inconsistent findings in the literature and the need for more rigorously designed studies to clarify the true nature of this relationship.

Overall, while a substantial number of studies, particularly prospective cohort studies, suggest that higher levels of physical activity are associated with a reduced risk of BPH and LUTS, the evidence is not entirely consistent. Methodological differences in defining BPH, assessing physical activity, and controlling confounding variables may contribute to the conflicting findings. Further well-designed prospective studies with objective measures of physical activity are needed to elucidate the complex relationship between exercise and BPH definitively.

### 3.3. Potential Biological Mechanisms Underlying the Protective Effects of Exercise Against BPH

While the precise mechanisms by which exercise may exert its protective effects and mitigate the risk and symptoms of BPH are still under investigation, current evidence suggests several key biological pathways are involved. Firstly, the modulation of sympathetic nervous system activity is implicated, as an overactive sympathetic tone can contribute to prostatic smooth muscle contraction and growth. Secondly, exercise induces beneficial alterations in hormonal profiles, potentially influencing the androgen-estrogen balance critical for prostate health. Thirdly, it leads to the suppression of inflammation and oxidative stress, both of which are recognized as significant contributors to BPH pathogenesis through cellular damage and proliferation. Finally, exercise is thought to promote autophagy within the prostate gland, a cellular process vital for clearing damaged components and maintaining tissue homeostasis, thereby potentially reducing uncontrolled cell growth.

#### 3.3.1. Influence on Sympathetic Nervous System Activity

BPH is characterized by a significant dynamic element of prostatic obstruction, which is influenced by increased smooth muscle tone in the prostate, a phenomenon largely driven by sympathetic nervous system activation. This sympathetic tone directly contributes significantly to urethral resistance, and medications like alpha-blockers, which reduce this tone, are highly effective in alleviating symptoms associated with BPH [48]. This pharmacological evidence strongly supports the role of sympathetic activity in BPH symptomatology. Elevated sympathetic autonomic activity has been correlated with higher scores on commonly used LUTS indices, such as the AUASI [49], further strengthening the clinical link between sympathetic tone and symptom severity. Notably, metabolic factors, such as energy intake, glycemic control, and adiposity, can influence sympathetic activity, with individuals maintaining a healthy weight generally exhibiting lower resting sympathetic activation compared to those with obesity [50]. This establishes a crucial mechanistic bridge, suggesting that lifestyle interventions impacting metabolic health could indirectly modulate sympathetic tone in the prostate. Epidemiological studies have also linked hyperinsulinemia and sedentary behavior to increased prostate growth, LUTS, and erectile dysfunction [51], reinforcing the interconnectedness of metabolic dysregulation, physical inactivity, and the pathophysiology of BPH and related conditions.

Given that exercise training is known to have a hypotensive effect and can reduce heightened sympathetic nervous system activity [52], sustained physical activity may offer a direct means of alleviating BPH and LUTS by reducing the tone of the enlarged prostatic smooth muscle. However, direct evidence demonstrated that regular exercise improves BPH/LUTS through direct or indirect modulation of prostatic sympathetic activity (e.g., via weight management or improved glucose metabolism) is currently lacking.

#### 3.3.2. Modulation of Hormonal Factors

Metabolic syndrome, a cluster of conditions including hypertension, diabetes, obesity, autonomic dysfunction, and dyslipidemia, is frequently and critically characterized by hyperinsulinemia, a key metabolic derangement [53]. This elevated insulin level is not merely a marker but a potential driver of prostate changes. The presence of metabolic syndrome has been significantly associated with larger initial prostate volumes and accelerated annual prostate growth rates [54,55], underscoring its direct impact on BPH progression. Furthermore, a substantial number of metabolic-syndrome-related factors are associated with BPH [56,57], indicating a complex interplay of metabolic disturbances. These findings are supported by studies indicating faster prostate growth in individuals with hyperinsulinemia compared to those without [58,59,60]. The proposed mechanism involves the proliferative effects of insulin on cells and the anti-apoptotic actions it mediates through insulin-like growth factor 1 (IGF-1) [58,59]. Thus, exercise-induced improvements in insulin sensitivity and metabolic health could indirectly mitigate BPH progression by normalizing these hormonal influences.

Androgens, known to play crucial roles in both BPH and prostate cancer development and progression, are also implicated in the etiology of BPH/LUTS [61]. Testosterone and its more potent metabolite, dihydrotestosterone (DHT), are crucial for the pathogenesis of BPH. 5-alpha-reductase inhibitors, a common treatment for BPH, work by blocking the conversion of testosterone to DHT, leading to a reduction in prostate size and significant alleviation of voiding symptoms [62]. Exercise, by influencing overall hormonal balance and metabolic factors, may indirectly modulate androgen metabolism, thereby impacting BPH progression.

#### 3.3.3. Attenuation of Inflammation and Oxidative Stress

Histological analyses of prostate tissue from BPH patients consistently reveal increased inflammatory responses, and the overexpression of pro-inflammatory cytokines within the prostatic microenvironment strongly suggests a significant role of inflammation in the development and progression of BPH [63]. This inflammatory state contributes to prostatic enlargement through direct proliferative signals and stromal remodeling. Inflammation is also recognized as a key driver in prostate cancer, highlighting a shared pathological mechanism in prostatic diseases. Furthermore, BPH has been linked to abnormal prostate growth induced by oxidative stress or IGF-1 signaling, both of which are exacerbated by inflammation. Consequently, suppressing inflammatory pathways represents a plausible strategy for reducing the risk of BPH. Regular physical activity is a known modulator of systemic inflammation and oxidative stress, offering a potential mechanism for its beneficial effects on prostate health.

Interestingly, observational studies have indicated a reduced risk of low urinary flow and prostate enlargement in men who regularly used nonsteroidal anti-inflammatory drugs (NSAIDs) or statins (cholesterol-lowering medications) [64,65,66]. These observations suggest a potential therapeutic avenue by targeting inflammation and lipid metabolism. However, these findings have not been consistently replicated in other large-scale cohort studies [67], suggesting that the relationship is complex and may be influenced by various confounding factors or specific patient populations. For example, Choi et al. reported higher levels of high-sensitivity C-reactive protein (hsCRP), a marker of systemic inflammation, in men with more severe LUTS, thus supporting a link between systemic inflammation and symptom burden; although another study found no such association [68], illustrating the inconsistency in inflammatory biomarker findings. The growing recognition of chronic prostatic inflammation and subsequent oxidative damage as contributing factors to the development of BPH has also highlighted the potential protective role of antioxidants [69], aligning with the broader understanding that mitigating cellular stress can prevent or slow BPH progression.

#### 3.3.4. Promotion of Autophagy

Emerging evidence suggests that autophagy, a fundamental cellular process involving the breakdown and recycling of intracellular components, may represent a novel therapeutic avenue for BPH [70]. This process is crucial for maintaining cellular health and preventing the accumulation of damaged organelles and proteins. Reduced autophagic activity has been observed in BPH tissues, suggesting that impaired cellular turnover due to decreased autophagy may directly contribute to the increased cellular proliferation characteristic of BPH [71,72]. Specifically, a decline in efficient waste removal can lead to cellular accumulation and uncontrolled growth, hallmarks of BPH.

Therefore, enhancing autophagy in BPH tissues to restore a balanced rate of cellular turnover could be therapeutically beneficial. While interventions like fasting and certain medications are known to induce autophagy, exercise has also been shown to activate this process in various tissues [73], offering a potential non-pharmacological strategy. Although direct evidence of exercise-induced autophagy in the prostate and its impact on BPH/LUTS is currently lacking, pharmacological studies offer suggestive insights. For instance, finasteride, a common BPH medication, has been shown to increase autophagy in prostate tissue, correlating with beneficial histological changes [71]. Furthermore, in animal models of BPH, drugs that activate autophagy have demonstrated the ability to alleviate BPH symptoms [72]. However, the precise mechanisms by which autophagy activation might alleviate BPH remain to be fully elucidated, requiring further investigation to translate these promising findings into clinical applications for BPH management.

### 3.4. Myokines and Their Role in Prostate Health

Myokines are diverse signaling molecules secreted by skeletal muscle during contraction, with autocrine, paracrine, and endocrine actions [74]. This broad range of action allows them to influence distant organs, as well as local muscle tissue. These molecules have been shown to influence a wide range of physiological processes, including inflammation, metabolism, and tissue remodeling [74,75]. Myokines, such as irisin, interleukin-6 (IL-6), and brain-derived neurotrophic factor (BDNF), are of particular interest in the context of BPH, as they may have direct or indirect effects on the prostate gland by modulating the very pathways implicated in its pathogenesis, such as inflammation and metabolism.

Irisin, a myokine released robustly in response to physical activity, has been shown to have anti-inflammatory and metabolic effects [7]. In animal models, irisin has been shown to reduce inflammation and promote fat browning, which may collectively influence prostatic tissue remodeling and reduce the risk of BPH [7]. Specifically, its anti-inflammatory properties could mitigate the chronic prostatic inflammation associated with BPH, while metabolic improvements might indirectly impact prostate growth. As physical activity reliably induces the release of irisin, its potential to counteract inflammation represents a compelling, although indirect, mechanism by which exercise could play a protective role in prostate health, thereby potentially decreasing the incidence or severity of BPH.

Interleukin-6 (IL-6), typically known as a proinflammatory cytokine, is another key myokine produced by skeletal muscle during exercise. While IL-6 has been implicated in the inflammatory processes associated with BPH pathogenesis, where its sustained high levels can promote prostatic growth and tissue remodeling, its role in exercise-induced inflammation is more nuanced. During physical activity, IL-6 is thought to exert an anti-inflammatory effect in the short term [10], acting as an exercise factor that can regulate systemic inflammatory responses. This temporary, beneficial action may help counterbalance the chronic inflammation that contributes to BPH progression. This dual nature of IL-6 suggests that, while it may be involved in the inflammatory pathways leading to BPH, its release during exercise might also have a beneficial, protective role.

Brain-derived neurotrophic factor (BDNF) is a neurotrophic factor with well-established effects on neuronal survival and plasticity. Exercise has been shown to significantly increase BDNF levels, which not only support cognitive health but also have anti-inflammatory effects and modulate metabolic functions [76]. BDNF may play a critical role in modulating the prostate’s response to inflammatory stimuli, potentially slowing the progression of BPH. This dual nature of IL-6 suggests that, while it may be involved in the detrimental chronic inflammatory pathways leading to BPH, its transient release during exercise might also have a beneficial, protective role by resolving or mitigating inflammatory cascades in the prostate and systemically [77].

Myostatin, a negative regulator of muscle growth, is another important myokine that is significantly influenced by physical activity. Regular exercise has been shown to reduce myostatin levels, which, in turn, confer widespread metabolic benefits by improving metabolic function through reducing insulin resistance, promoting fat breakdown, and increasing the expression of adiponectin and irisin [8,9]. These metabolic benefits not only contribute to overall health but may also have a significant impact on prostate health by ameliorating metabolic syndrome components that drive BPH. Myostatin’s role in regulating cellular growth and differentiation could influence prostate tissue remodeling and the development of LUTS associated with BPH. For instance, its inhibitory effects on cell proliferation could, if dysregulated, contribute to abnormal prostate growth. Although the exact mechanisms linking myostatin to BPH remain under investigation, reducing myostatin through exercise could serve as a protective strategy, helping to reduce inflammation, improve metabolic functions, and mitigate the pathological processes involved in BPH. Further research is needed to fully delineate the specific signaling pathways through which myostatin impacts prostate tissue.

Together, these myokines—irisin, IL-6, BDNF, and myostatin—highlight the complex and multifaceted relationship between exercise, inflammation, metabolism, and prostate health. As research into the role of myokines in BPH continues, it is becoming increasingly clear that regular physical activity may offer a non-pharmacological approach to preventing and managing BPH, potentially providing a beneficial strategy to reduce the risk and progression of this common condition. Further exploration of myokine mechanisms will be essential to identify potential therapeutic targets and refine exercise-based interventions for prostate health.

## 4. Strengths and Limitations

This narrative review offers a comprehensive synthesis of current evidence exploring the potential role of physical activity and myokines in the modulation of BPH. One of the key strengths of this review is its interdisciplinary approach, integrating insights from urology, endocrinology, and exercise physiology to provide a novel perspective on the non-pharmacological management of BPH. The inclusion of both supportive and contradictory findings from epidemiological and mechanistic studies enhances the objectivity and transparency of the discussion. Furthermore, the review categorizes the available evidence by mechanisms—such as sympathetic tone regulation, hormonal modulation, inflammation suppression, and autophagy promotion—thus offering a structured framework for understanding the multifactorial impact of exercise on prostate health.

However, several limitations must be acknowledged. As a narrative review, this work is inherently limited by the potential for selection bias, as study inclusion was not based on a systematic search protocol. The heterogeneity in the definition and measurement of physical activity across studies also makes direct comparison challenging. Furthermore, while this review proposes myokines as a biologically plausible mechanistic link between skeletal muscle activity and prostate health, there is a notable absence of direct clinical data demonstrating causal effects of specific myokines on prostatic tissue remodeling in humans. Most mechanistic insights regarding myokines are derived from preclinical models or inferred from studies on other organ systems, limiting the strength of inference in the context of BPH. Additionally, potential publication bias and the predominance of observational data may affect the generalizability of conclusions. Future research should prioritize mechanistic human studies and well-designed clinical trials to validate these hypotheses and further clarify the role of exercise-mediated signaling pathways in BPH progression.

## 5. Conclusions

BPH is a complex, multifactorial disease that affects aging men and can significantly impact quality of life. While hormonal and inflammatory factors have long been recognized as key contributors to BPH, emerging research suggests that skeletal muscle and myokines may also play important roles in modulating prostate health. Regular physical activity, through the release of myokines, may protect against BPH by reducing inflammation, improving metabolic function, and influencing prostatic tissue remodeling. Although direct evidence linking myokines to BPH is limited, the available data suggest that myokines may play a key role in modulating inflammation and metabolic functions, both of which are central to the pathophysiology of BPH. As our understanding of the myokine–exercise–prostate connection deepens, exercise-based interventions may provide a promising non-pharmacological approach to the prevention and management of BPH. Further research is needed to clarify the specific myokines involved in this process and to explore their mechanisms of action in the context of prostate health. Future studies should focus on identifying these myokines and examining their potential as therapeutic targets for BPH.

## 6. Future Directions

To advance our understanding of the exercise–myokine–prostate axis, future clinical trials are warranted. A prospective randomized controlled trial (RCT) framework could be designed to evaluate the impact of structured exercise interventions (e.g., moderate-intensity aerobic training or resistance exercise) on prostate volume, LUTS severity, and circulating myokine levels (such as irisin, IL-6, myostatin, and BDNF) in men with early-stage BPH. Baseline and follow-up assessments should include digital rectal exams, prostate imaging (e.g., transrectal ultrasound or MRI), validated symptom scores (e.g., IPSS), and biomarker assays for systemic inflammation and hormonal profiles. An ideal trial would include a sedentary control group, an exercise-only group, and potentially an exercise-plus-diet modification arm to control for confounders. In parallel, mechanistic sub-studies using prostate tissue (e.g., from patients undergoing surgical intervention) could explore receptor expression or signaling pathway alterations associated with myokine exposure. Such a framework would help elucidate causal links and identify therapeutic exercise “doses” that are most effective in modulating prostate health.

## Figures and Tables

**Table 1:** Summary of studies provides evidence on the relationship between physical activity and BPH.

Study	Study Design	Number of Participants	Reported Conditions	Key Findings
Platz et al., 1998 [38]	Prospective Cohort	30,634	BPH/LUTS	A significant inverse relationship was observed between physical activity and BPH risk, with individuals in the highest quintile of exercise exposure (≥33.8 MET∙hr/week) demonstrating a 25% reduced likelihood of requiring BPH-related surgery compared to those in the lowest quintile (0.1–3 MET∙hr/week), reflected by an odds ratio of 0.75. This finding suggests that increased physical activity may confer a protective effect against clinically significant BPH.
Meigs et al., 2001 [39]	Cohort Study	1019	BPH	An odds ratio of 0.5 was observed when comparing individuals in the highest quartile of physical activity to those in the lowest quartile, indicating that men with the highest exercise levels had a 50% lower risk of developing BPH. This finding supports a strong inverse association between physical activity and BPH incidence.
Lacey et al., 2001 [37]	Case–Control Study	677	BPH	No statistically significant association was identified between physical activity levels and the risk of developing BPH in this study, suggesting that exercise may not uniformly influence BPH outcomes across all populations or study designs.
Prezioso et al., 2001 [40]	Cohort Study	1033	LUTS	Individuals engaging in regular physical activity demonstrated a lower risk of experiencing specific LUTS, including incomplete bladder emptying, urinary intermittency, urgency, and repeated voiding, suggesting a protective association between exercise and the severity of BPH-related symptoms.
Joseph et al., 2003 [41]	Case–Control Study	708	LUTS	After adjusting for potential confounders, such as age, BMI, and comorbidities, the association between physical activity and BPH risk was no longer statistically significant, indicating that the initially observed relationship may be influenced by other underlying factors.
Rohrmann et al., 2005 [42]	Case–Control Study	2797	LUTS	Physically active individuals had a 52% lower odds of developing lower urinary tract symptoms (LUTS) compared to their sedentary counterparts (OR = 0.48), indicating a strong inverse association between regular physical activity and BPH-related symptom severity.
Dal Maso et al., 2006 [36]	Case–Control Study	2820	BPH	The odds of developing BPH were reduced by 30% to 40% among individuals engaging in higher levels of occupational physical activity (OR = 0.6–0.7), and by 30% to 50% among those reporting greater levels of recreational physical activity (OR = 0.5–0.7), highlighting a dose-dependent inverse relationship between physical activity and BPH risk.
Hong et al., 2006 [43]	Cross-Sectional Study	641	BPH/LUTS	While no significant overall difference was found between exercising and non-exercising groups, men who engaged in moderate levels of exercise demonstrated a reduced odds of BPH compared to those with low or no exercise, suggesting that moderate physical activity may confer protective benefits.
Parsons et al., 2008 [14]	Systematic review and meta-analysis	43,083	NA	The odds ratios for BPH ranged from 0.70 to 0.74 across individuals reporting low to high levels of physical activity, indicating a consistent, modest inverse association between physical activity and BPH risk.
Kristal et al., 2007 [35]	Cohort Study	5667	LUTS	Although no statistically significant association was observed between physical activity levels and BPH risk, the data suggest a possible trend without clear evidence supporting a protective effect of increased physical activity.
Williams PT. 2008 [44]	Cohort Study	1899	BPH	The odds ratio of 0.68 comparing the fastest to the slowest exercisers indicates that higher exercise intensity is associated with a 32% reduced risk of BPH, suggesting a protective effect of more vigorous physical activity.
Lagiou et al., 2008 [45]	Case–Control Study	430	BPH	The odds ratio of 0.59 for individuals with high versus low levels of physical activity indicates that those engaging in higher physical activity have a 41% lower risk of developing BPH, highlighting the protective association between increased exercise and BPH risk.
Lee et al., 2014 [46]	Cohort Study	582	BPH	The odds ratio of 0.93 for individuals with lower sedentary time suggests a modest reduction in BPH risk, whereas an odds ratio of 0.17 for those with higher sedentary time indicates a substantially increased risk, highlighting the detrimental impact of prolonged sedentary behavior on BPH development.
Wolin et al., 2015 [47]	Cohort Study	4710	LUTS	The odds ratio decreased to 0.87 for nocturia and further to 0.66 for severe nocturia among men engaging in regular physical activity, indicating that exercise is associated with a 13% reduction in the risk of experiencing nocturia and a 34% reduction in the risk of severe nocturia.
Jia et al., 2024 [12]	A Two-Sample Mendelian Randomization Study	NR	NR	In this study, no significant association was found between sedentary behavior or varying levels of physical activity and the risk of developing BPH, indicating that neither increased exercise nor sedentary time alone significantly influenced BPH risk within the study population.

BPH: benign prostatic hyperplasia; LUTS: lower urinary tract symptoms; NR: not reported; NA: not applicable.

## Abbreviations

The following abbreviations are used in this manuscript:

BPH	Benign prostatic hyperplasia
LUTS	Lower urinary tract symptoms
BDNF	Brain-derived neurotrophic factor
IL-6	Interleukin-6
